# Effect of Integrated Naturopathy Interventions on Systemic Inflammatory Markers and Quality of Life in Patients With Active Rheumatoid Arthritis: A Randomized Controlled Trial

**DOI:** 10.7759/cureus.57764

**Published:** 2024-04-07

**Authors:** Geetha B Shetty, Prashanth Shetty, Balakrishna Shetty

**Affiliations:** 1 Acupuncture and Energy Medicine, Sri Dharmasthala Manjunatheshwara (SDM) College of Naturopathy and Yogic Sciences, Ujire, IND; 2 Yoga, Sri Dharmasthala Manjunatheshwara (SDM) College of Naturopathy and Yogic Sciences, Ujire, IND; 3 Biochemistry, Sri Dharmasthala Manjunatheshwara (SDM) College of Naturopathy and Yogic Sciences, Ujire, IND

**Keywords:** rheumatoid factor, interleukin-6, acupuncture, inflammatory markers, naturopathy, rheumatoid arthritis

## Abstract

Background: Rheumatoid arthritis (RA) is a chronic, systemic, polyarticular autoimmune inflammatory disease that destroys the capsule and synovial lining of joints. Antirheumatic treatment reduces disease activity and inflammation, but not all patients respond to treatment. Naturopathy, a research-based complementary and alternative medicine, may be useful in these patients, but there is little data on the effect of Naturopathy interventions on inflammation and disease activity in RA.

Objective: To explore the effect of 12 weeks of integrated naturopathy interventions on disease-specific inflammatory markers and quality of life in RA patients.

Methods: A total of 100 RA patients were randomized into two groups: the naturopathy group (integrated naturopathy interventions with routine medical therapy) and the control group (only with routine medical therapy). Blood samples were collected pre- and post-intervention for primary outcome measurements of systemic inflammatory markers (ESR, CRP, and IL-6). Disease activity score (DAS-28) and quality of life were used to assess disease activity and functional status using SF-36, respectively, at pre- and post-intervention time points.

Results: The results of the present study show a notable decrease in disease activity after 12 weeks of naturopathy intervention. As such, a significant decrease was found in levels of systemic inflammatory markers such as ESR (p = 0.003) and IL-6 (p < 0.001), RA disease activity score (DAS-28) (p = 0.02), and most of the components of health-related quality of life (SF 36 scores) (p < 0.05) except in vitality (p = 0.06).

Conclusions: The findings of the present study suggest that integrated naturopathy treatments may have the ability to control persistent inflammation, maintain immune homeostasis, and lower disease activity.

## Introduction

Rheumatoid arthritis (RA) is a systemic chronic inflammatory autoimmune disease characterized by inflammatory arthritis and extra-articular involvement primarily involving synovial joints [1]. The prevalence of RA ranges from 0.24% to 1% of the world population [2] and 0.7% in the Indian population [3]. Prevalence and incidence rates in women are twice as high compared to men, affecting all age groups, but peak incidence is noted between 50 and 60 years of age [4]. Predisposing genetic factors, along with factors such as dietary habits, nicotine consumption, physical activity, and socioeconomic status, influence the condition of the patient [5]. Pharmacological treatments for RA include nonsteroidal anti-inflammatory drugs (NSAIDs), glucocorticoids (GC), and disease-modifying antirheumatic drugs, along with analgesics [6]. Even with a well-formed treatment protocol, RA seems to affect different aspects of being, especially emotional and social well-being, which contribute to persistent inflammation. Hence, the addition of non-pharmacological therapies (e.g., hydrotherapy, mud therapy, massage therapy, and acupuncture) fulfills the treatment goals of RA.

Naturopathic medicine is a mode of primary healthcare, which is defined as a drugless, noninvasive, and evidence-based system of medicine relaying natural elements based on theories of vitality, toxemia, and self-healing [7]. Naturopathy, a research-based complementary and alternative medicine (CAM), includes various lifestyle and behavioral changes, dietary modifications including time-restricted feeding, and manual therapies such as hydrotherapy, mud therapy, massage therapy, and so on [7]. Studies state that inflammation present in the body can be readily reduced using dietary modifications [8] and other manual therapies where rheumatic symptoms seem to improve individually. Naturopathy as a potent non-pharmacological treatment offers the same in an integrative form, where only case reports have been published on the management of RA, which showed significant improvement in pain symptoms, reduction in ESR, RA factor, and anti-cyclic citrullinated peptide (CCP) [9] and quality of life in various diseases [10]. Studies focusing on naturopathy and systemic inflammation are difficult to find. Hence, this 12-week study of integrated naturopathy interventions evaluates their impact on systemic inflammatory markers and quality of life in RA.

## Materials and methods

Study design

This study was a randomized, parallel-group, active-controlled trial to evaluate the effectiveness of 12 weeks of integrated naturopathy intervention on RA patients under disease-modifying antirheumatic drugs (DMARDs).

Participants

Study participants (n = 100) were selected from the Sri Dharmasthala Manjunatheshwara (SDM) Hospital outpatient department from December 2020 to June 2022. The inclusion criteria of the study include RA patients with a DAS 28 score greater than 2.6 (diagnosed as per 2010 American College of Rheumatology/European League Against Rheumatism (ACR/EULAR) RA classification criteria), aged 18-60 years, and who were on routine medical treatment for at least six months. Pregnant or nursing mothers, people with any other autoimmune disease, and people with a history of recent oral or intra-articular steroid use within the previous six months were all excluded.

Ethical considerations

The study was started after acquiring ethical clearance (IEC-198/17.10.2018) from the Institutional Ethics Committee and registration under the clinical trials registry, India (CTRI/2020/12/029833).

Sample size

Using the G power software, the sample size was determined by setting the alpha at 0.05 and powering it at 0.8 for a medium effect size (according to Cohen’s d), considering the mean and SD of an earlier study [7] and assuming a 15-20% attrition rate. For each group, we chose to enlist 50 participants (for a total of 100).

Randomization

**Figure 1:**
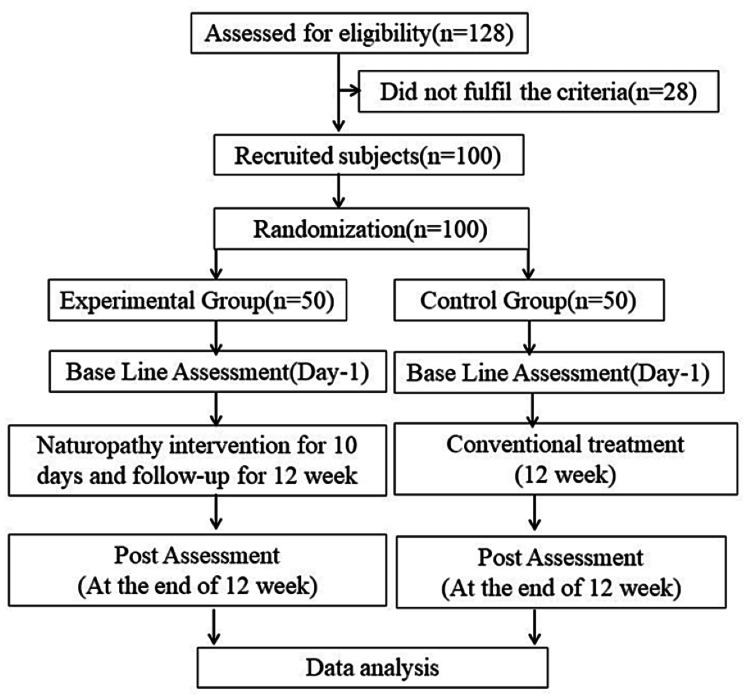
Trial profile

The software http://www.randomization.com was used to randomly divide the 100 participants into two groups. Using this randomization technique, 50 participants were assigned to each group. As a result, this research uses a parallel group design with a 1:1 allocation ratio. Group 2 was designated as the control group and was given regular medication, while Group 1 underwent a 12-week naturopathy intervention. The trial profile's Consolidated Standards of Reporting Trials (CONSORT) flowchart is displayed in Figure 1.

Intervention

Details of the integrated naturopathy intervention and dietary intervention given to the patients of the experimental group for 10 days are shown in Tables 1-2, respectively.

**Table 1:** Details of integrated naturopathy intervention given to the patients in the experimental group Note: *: bilateral needling; ¶’: single needling; GB: gallbladder; GV: governing vessel; LI: large intestine; UB, urinary bladder; SP: spleen; ST: stomach; EX: extraordinary points

Therapy	Specific treatment	Duration/session (min)	Total number of sessions/10 days
Hydrotherapy	Alternate hot and cold-water application to the painful joints	11 min	8
	Sauna bath	10–15 min	3
	Enema (during fasting)	10 min	5
Mud therapy	Mud application to the painful joints(40º–45ºC)	25–30 min	8
Massage therapy	Partial massage to bilateral hands and legs	30 min	2
	Full-body massage	45 min	2
Acupuncture (Needling points)	LI-4*, LI-11*, UB-11*, GB-34*, SP-6*, ST-44*, GV-20*, EX-28’, EX-36’	30 min	10

**Table 2:** Detail of diet intervention given to the patients in the experimental group

Sl. No	Days	Diet content	Calorific value
1	Day 1	Naturopathic boiled diet, three times daily	1,500–2,000 Kcal
2	Day 2	Raw diet inclusive of sprouts, fruits, or vegetable salad, three times daily	1,500 Kcal
3	Day 3	Fruit or vegetable diet, three times daily	1,000 Kcal
4	Days 4, 5, and 6	Carrot juice 250 mL, four times daily	600 Kcal
5	Day 7	Apple juice 250 mL, four times daily	800 Kcal
6	Day 8	Fruit or vegetable diet, three times daily	1,000 Kcal
7	Day 9	Fruit or vegetable diet, three times daily	1,000 Kcal
8	Day 10	Raw diet inclusive of sprouts, fruits, or vegetable salad, three times daily	1,000 Kcal

Then the subjects were followed for 12 weeks post intervention at home with alternate hot- and cold-water applications to the painful joints and a prescribed naturopathic diet.

Outcome measures

Assessments at baseline and the end of the 12th week included clinical and laboratory parameters. Clinical assessment included a visual analog scale (VAS) for pain (0 indicating no pain and 10 indicating the worst pain imaginable), the number of swollen and tender joints, and a patient global assessment (PGA) for overall health (by asking a single question: “Considering all the ways your arthritis has affected you, how do you feel about your health status at this time?”. Responses were collected by asking to mark 0-100 on a numeric rating scale (0 = very good and 100 = very poor). To assess the quality of health-related quality of life, a short form-36 version 2 (SF-36 v2) health survey, a 36-item questionnaire that measures health in eight dimensions, was used [11]. Laboratory assays, including ESR, CRP, interleukin-6, rheumatoid factor (RA factor), and C-reactive protein, were performed.

Statistical analysis

The Shapiro-Wilk test for normality was utilized to evaluate the distribution of the research variables for normalcy. The study variables were found to be non-normally distributed. A Mann-Whitney U test was employed to compare the differences between the study groups (i.e., naturopathy intervention group vs. control group). A P value of < 0.05 was considered statistically significant.

## Results

The present study was conducted to find out the outcomes of a 12-week integrated naturopathy intervention on RA individuals. The characteristics of the study sample are shown in Table 3.

**Table 3:** Characteristics of the study participants Height, BMI (body mass index), age, and duration of disease data are expressed as mean±SD

Sl. No	Variables		Naturopathy (n = 50)	Control (n = 50)
1	Gender	Female	35	33
		Male	15	17
2	Height (cm)		160.68±9.02	156.70±8.10
3	BMI (kg/m^2^)		28.4±4.30	26.9±3.47
4	Age (yr)		52.64±10.43	47.80±9.27
5	Duration of disease (yr)		5.79±3.08	6.07±2.59

The baseline was comparable, and there was no significant difference between the study and control groups. The group comparison of systemic inflammatory markers (ESR, CRP, and IL-6) and RA disease-specific markers showed significant changes (p<0.05) in ESR, DAS-28, and IL-6 (Table 4).

**Table 4:** Comparison of systemic inflammatory markers (ESR, CRP, and IL-6) and RA disease-specific markers at the 12th week post intervention Data are expressed as mean±SD. Abbreviations: ESR: erythrocyte sedimentation rate, CRP: C-reactive protein, DAS 28: disease activity score, IL-6: interleukin-6, RF: rheumatoid factor, PGA: patient global assessment, VAS: visual analog scale for pain intensity). The statistical test used was the Mann-Whitney U test. Level of significance: *= p<0.05 was considered significant.

Sl. No	Study variables	Assessment	Naturopathy group (n = 50)	Control group (n = 50)	P value
1	ESR (mm/hr)	Baseline	54.60±11.48	57.02±15.54	
		Post-intervention	39.22±13.34	48.20±15.49	0.003*
2	CRP (mg/L)	Baseline	35.68±32.26	38.24±34.38	
		Post-intervention	28.05±29.13	33.02±30.11	0.134
3	DAS-28	Baseline	4.81±0.65	4.25±0.83	
		Post-intervention	3.59±0.75	3.84±0.69	0.021*
4	RF (IU/mL)	Baseline	57.02±28.01	39.02±28.01	
		Post-intervention	46.53±22.65	38.14±23.81	0.114
5	IL6 (pg/mL)	Baseline	72.73±55.07	61.25±52.83	
		Post-intervention	44.26±41.46	57.8±39.86	<0.001*
6	PGA	Baseline	55.22±12.12	49.54±12.34	
		Post-intervention	43.46±12.41	45.66±13.24	<0.001*
7	VAS	Baseline	3.60 (±1.26)	5.10 (±1.09)	
		Post-intervention	3.12 (±1.29)	4.68 (±0.82)	<0.001*

The pain intensity that was measured on a numerical rating scale, with scores ranging from 0 to 10, also showed significant changes (p<0.05) between the groups. The comparison of health-related quality of life (SF-36 scores) in the naturopathy vs. control groups showed significant changes (p<0.05) in all components except vitality (Table 5).

**Table 5:** Comparison of health-related quality of life (SF-36 scores) at the 12th week post intervention Data are expressed as mean±SD. Abbreviations: physical functioning (PF), role function (RP) (physical aspect), bodily pain (BP), general health perception (GH), mental health (MH), role function (emotional aspect), short form (SF), vitality (VT), physical component summary scores (PCS), mental component summary score (MCS). The statistical test used was the Mann-Whitney U test. Level of significance: *= p<0.05 was significant.

Sl. No	Study variables	Assessment	Naturopathy group (n = 50)	Control group (n=50)	P value
1	PF	Baseline	38.51±10.65	36.84±10.69	
		Post-intervention	42.59±12.75	33.25±11.83	<0.001*
2	RP	Baseline	29.51±12.45	29.22±12.88	
		Post-intervention	38.22±11.28	36.26±11.20	<0.001*
3	BP	Baseline	30.01±11.26	37.62±12.08	
		Post-intervention	36.36±13.77	31.45±11.55	0.023*
4	GH	Baseline	43.46±12.41	45.66±13.24	
		Post-intervention	55.22±12.12	49.54±12.38	0.021*
5	MH	Baseline	51.02±17.32	44.92±10.27	
		Post-intervention	58.02±16.52	44.92±12.67	<0.001*
6	RE	Baseline	33.46±14.42	35.66±16.24	
		Post-intervention	35.22±15.36	37.54±13.62	0.046*
7	SF	Baseline	48.66±12.41	45.36±13.24	
		Post-intervention	55.22±12.12	49.54±12.38	<0.001*
8	VT	Baseline	43.46±22.42	41.66±18.74	
		Post-intervention	52.22±16.62	49.54±14.38	0.063
9	SF-36 PCS	Baseline	32.23±14.42	34.86±10.24	
		Post-intervention	36.29±11.41	36.06±12.28	0.014*
10	SF-36 SCS	Baseline	37.23±15.45	37.86±13.28	
		Post-intervention	40.11±15.34	39.86±16.08	0.005*

## Discussion

Short-term naturopathic interventions for 12 weeks, including hydrotherapy, mud therapy, massage, acupuncture, and diet therapy had greater acceptance among RA patients and were implemented in a hospital setting as per the protocol. The study's findings demonstrated a significant drop in DAS-28 and inflammatory indicators, including IL-6 and ESR, between the pre- and post-intervention periods. Thus, short-term naturopathic intervention, when used as a treatment modality, significantly lowers systemic inflammation, disease activity, and progression in patients with RA. It can also be used as a powerful adjunct therapy in compliance with National Institute for Health and Care Excellence (NICE) guidelines for the non-pharmacological treatment of RA [12].

The hydrotherapeutic applications based on naturopathy are responsible for the observed decrease in pain in the study patients. It is commonly anticipated that thermotherapy will raise tissue temperature, promote local blood flow, increase soft tissue flexibility, induce local vasodilation, raise metabolite production, and lessen pain by lowering muscular spasms [13].

Research has demonstrated that by encouraging muscular relaxation, massage therapy also lessens pain. According to our findings, massage therapy may encourage parasympathetic activation, which lowers stress and cortisol and increases serotonin, dopamine, and endorphins. These chemicals are helpful for reducing pain and improving the quality of sleep [14].

One possible underlying mechanism for pain alleviation is the stimulation of pressure receptors, which leads to an increase in vagal activity and serotonin levels (the body's natural pain suppressor). Significant increases in vagal activity and serotonin levels and a reduction in the level of substance P have occurred after massage therapy [15]. By acting on thermal receptors and mechanoreceptors, the temperature and pressure of the water used in hydrotherapy can block nociceptors and have a beneficial effect on spinal segmental processes, which is beneficial for painful conditions [9].

Our study findings reported a significant reduction of IL-6, a proinflammatory pleiotropic cytokine whose persistent secretion is involved in chronic inflammation through its peculiar signaling pathway using a specific receptor, IL-6R, and co-receptor glycoprotein gp130 [16]. Through its reduction, short-term naturopathic interventions might have improved the relative population of regulatory T cells (Treg) cells to T helper 17 (Th17), hence the immune homeostasis. Moreover, IL-6 triggers the production of acute-phase proteins, which include CRP, activation of the hypothalamic-pituitary-adrenal (HPA) axis, and stimulation of B cells to produce autoantibodies, which contribute to systemic inflammation, fatigue, and symptoms worsening [17]. Our present study findings suggest a possible inhibition of these mechanisms. Moreover, mud therapy is known to reduce inflammation, and sulfur minerals present in the mud absorbed by the skin might cause an analgesic effect [18].

Dong et al. indicated that the toll-like receptor (TLR) signaling pathway contributed to the development and progression of RA. 18 Acupuncture could reduce the expression of TLR4, thus leading to anti-inflammatory effects [19]. Studies have also shown that acupuncture can lower tumor necrosis factor-alpha (TNF-α) and vascular endothelial growth factor (VEGF) in peripheral blood and joint synovia to improve the internal environment, which is beneficial for RA [20]. Another possible mechanism could be attributed to the antioxidative effect, such as inducing the increased activities of superoxide dismutase (SOD) and catalase in the serum of RA. This alleviates oxidative stress and inflammation and triggers the release of endorphins found after acupuncture in RA patients [21]. Moreover, studies show that fasting [22] and a rich antioxidant diet can reduce inflammation by regulating oxidative stress [23]. Studies have shown that quality of life is comparatively low in all domains (physical health, mental health, and emotional aspects) in RA patients because of fatigue, pain, stiffness, and impaired physical functioning [24]. Our study has shown significant improvement in all domains of SF-36 and VAS score, which explains the potential of naturopathic intervention as a non-pharmacological management for RA.

Limitations

During the follow-up period at home, there was no direct supervision of the participants regarding their naturopathic and diet intervention, despite each day's food intake being recorded by the subjects.

The patients included in the control group who were not regular with the medication before the study started taking it regularly after hearing the RA complications addressed while obtaining consent from the participants, which may have an impact on the results.

The present study was conducted in the short term. For a better understanding of the mechanisms behind the efficacy of naturopathic treatment in RA, long-term interventions may be warranted.

## Conclusions

The findings of the present study suggest that naturopathic treatments can control persistent inflammation, maintain immune homeostasis, and lower disease activity. These results imply that short-term naturopathic lifestyle intervention may affect the pathobiology of RA by focusing on systemic inflammation, resulting in its remission and improvement in physical symptoms.
